# Neonatal Pain Management: Is There An Endocrinal Response?

**DOI:** 10.2174/0118715303325556241127080237

**Published:** 2025-01-08

**Authors:** Mohamed Shawky Elfarargy, Ahmad Roshdy Ahmad, Dalia Hamdy Elbadry

**Affiliations:** 1 Department of Pediatrics, College of Medicine, Jouf University, Sakaka, KSA;; 2 Department of Pediatrics, Tanta University, Tanta, Egypt;; 3 Department of Pediatrics, El Menshawy General Hospital, Tanta, Egypt

**Keywords:** Neonate, endocrine, opioids, acetaminophen, breast milk, sucrose, non-nutritive sucking, analgesic drugs, anesthetic drugs

## Abstract

Neonates exhibit pain responses characterized by various endocrinal changes, including alterations in cortisone and oxytocin serum levels, as well as physiological and emotional reactions. The administration of neonatal pain management leads to the normalization of endocrine hormones, including cortisone and oxytocin, which are affected by the presence of neonatal pain. Diagnosing neonatal pain is complex; however, effective management is essential. An adequate balance should be established between the analgesics used for pain management and their associated side effects. Uncontrolled neonatal pain is correlated with delayed development with increased neurologic insult. This review aims to examine the significance of neonatal pain, along with its clinical and physical manifestations. It also explores strategies for managing neonatal pain, encompassing both pharmacological and non-pharmacological methods, along with the particular medications utilized in pharmacological interventions. This discussion includes various non-pharmacological methods for managing neonatal pain. Additionally, this review examines methods for pain assessment. The aim is to highlight the significance of pain in this vulnerable population and to promote the implementation of diverse management strategies for neonatal pain to prevent serious yet avoidable, adverse effects in neonates.

## 1 INTRODUCTION

### 1.1 Definition

Pain is a distressing emotional and sensory experience, and the inability to convey these sensations to others does not imply their absence. Identifying pain in newborns poses significant challenges, often leading to a lack of appropriate treatment. Neonates undergo various painful procedures in the neonatal intensive care unit, where healthcare professionals possess specialized training and equipment to provide optimal care for infants. The painful procedures include heel lancing, cannulation, NG insertion, blood sampling, and catheterization. Neonatal pain manifestations encompass facial grimacing, alterations in vital signs (such as oxygen desaturation and increased heart rate), and prolonged healing times. The long-term effects of unmanaged pain include disturbed emotional relations, impaired mental and social development, and neurologic insult. The presence of various harmful effects from untreated pain in newborns underscores the significance of proper management [1-4].

### 1.2 Endocrinal Responses to Neonatal Pain

Neonatal pain may cause a reduction in cortisol response and levels, as cortisol is considered the primary stress hormone in humans. Hormonal changes associated with neonatal pain management, whether through medical or nonmedical methods, have been observed to result in increased oxytocin levels corresponding to reduced pain sensation. Oxytocin is a hormone associated with attachment, secreted in the hippocampus, and plays a vital role in pain response. It may also offer protection against inflammation that can occur in the brain of a preterm neonate [5-9].

### 1.3 Importance of Neonatal Pain

Furthermore, unmanaged pain in neonates can lead to prolonged effects, such as impaired emotional relationships, hindered mental and social development, and neurological damage, given that pain exposure occurs during critical periods of nervous system development. Pre-term neonates were found to demonstrate an increased pain response in comparison to full-term neonates, as well as diminished emotional relationships, impaired mental and social development, and neurologic deficits relative to full or post-term neonates. The management of neonatal pain is crucial, as it enhances physiological, neurological, and social outcomes. Pain assessment in neonates presents challenges, as they are unable to articulate their pain experiences. Pain assessment in neonates presents challenges due to their inability to articulate pain sensations. Although various pain scoring systems exist, none has been universally validated as the sole measure for neonatal pain assessment. Furthermore, our understanding of the mechanisms underlying pain sensation in neonates is insufficient. Many neonatal centers fail to address neonatal pain and lack protocols for its management, leading to various adverse effects associated with uncontrolled neonatal pain [10-14].

### 1.4 Neonatal Pain Scores

The (Caring, Observant, Mindful, Friendly, Obliging, Responsible, Tactful) COMFORTneo score is a pain assessment tool that has been validated for neonates, particularly premature neonates. This score is composed of six parameters: alertness, cry or breathing, body motion, calmness or agitation, muscle tone, and facial expression. Each parameter is assigned a value between 1 and 5 depending on the neonate’s response. A COMFORTneo score below 14 indicates effective pain control, scores ranging from 14 to 21 reflect moderate pain, and scores between 22 and 30 signify severe pain. The Neonatal Infant Pain Score (NIPS) is a pain assessment tool for neonates, comprising parameters, such as crying, facial expression, respiratory patterns, upper limb movement, lower limb movement, and alertness, yielding a maximum score of 7. Each parameter is assigned a value of 1 if present, except for the crying parameter, which received a maximum score of 2 [15-29].

### 1.5 Management of Neonatal Pain

The intervention comprises pharmacological measures, including both opioid and non-opioid medications, such as acetaminophen, NSAIDs (*e.g.,* ketorolac, ibuprofen), dexmedetomidine, and gabapentin. Additionally, it incorporates non-pharmacological therapies and environmental measures, such as oral sucrose/glucose and facilitated tucking, as demonstrated in Fig. (**1**) [30-55].

#### 1.5.1 Pharmacological Measures

##### 1.5.1.1 Opioid Drugs

Opioid analgesics are the most effective therapy for managing moderate to severe pain in neonates, providing both analgesia and sedation. Opioids are regarded as an effective method for pain management in neonates. Morphine may be utilized as a first-line medication for postoperative pain management in neonates, while parenteral opioids can be administered to intubated neonates. Opioid overuse presents numerous risks, including adverse neurodevelopmental outcomes, mortality, extended hospitalization, and the potential for addiction or habituation. Adverse effects of opioids are more prevalent and severe in premature infants. Opioids are associated with several adverse effects, including decreased blood pressure, reduced gastrointestinal and urinary motility, decreased heart rate, and, specifically for fentanyl, chest wall rigidity. Remifentanil, a derivative of fentanyl, is characterized by its short-acting properties and potential application in minor procedures; however, its use remains a subject of controversy. Opioids may be administered in various dosages and can alleviate the stress response associated with pain in neonates. Morphine and fentanyl are regarded as the most significant opioids in use [56-60].

**i-Morphine**

Morphine is a popular opioid utilized for neonatal pain management. It is primarily administered as a continuous intravenous infusion in ventilated or postoperative neonates to alleviate severe pain associated with invasive neonatal procedures. It has been validated for effectiveness and safety in these indications; however, it remains under active investigation to monitor the results of its use regarding effectiveness and safety. Morphine enhances ventilator synchronization in neonates receiving mechanical ventilation. Morphine does not increase the risk of mortality, intracranial hemorrhage, or brain atrophy while effectively reducing pain scores with only a slight increase in heart and respiratory rates. Other adverse effects include a decrease in blood pressure among ventilated neonates, as well as prolonged duration of mechanical ventilation and delayed tolerance of enteral feeding. Morphine significantly reduces pain in neonates, particularly those on mechanical ventilation, without evidence of increased mortality or heightened risk of BPD, NEC, or IVH. However, adverse effects, such as delayed feeding tolerance, have been observed in preterm infants. The impact of morphine administration on neurodevelopmental outcomes in this population remains unproven and contentious. Some neonates exhibited improved neurological development when administered morphine prior to painful, invasive procedures compared to those who did not receive morphine. This improvement may be attributed to the potential impact of unmanaged pain from such procedures on the neurodevelopmental outcomes of these neonates. The administration of morphine *via* intravenous infusions in ventilated neonates offers numerous advantages, particularly as these patients undergo various painful, invasive procedures, such as endotracheal tube insertion, intravenous line placement, and frequent sampling for monitoring, as well as chest tube insertion in cases of pneumothorax. Morphine is regarded as safe and effective for managing postoperative pain in full-term neonates, according to numerous studies. Nevertheless, the safety of morphine administration in preterm neonates remains unexamined. The effectiveness of morphine as an analgesic in alleviating neonatal pain, along with its safety and potential side effects on newborns, remains controversial [61-71].

**ii-Fentanyl**

Fentanyl produces rapid analgesic effects along with minimal adverse effects in neonates. Fentanyl decreases the stress hormone in the blood. Research indicates that pain scores and cytokine levels after painful heel sticks significantly decreased following fentanyl administration. Fentanyl (1-2 mcg/kg) is primarily utilized for analgesia prior to painful procedures in both full-term and preterm neonates. Fentanyl infusions have been shown to reduce pain scores while minimizing sedation and hypotension effects. Additionally, fentanyl exhibits a decreased impact on intestinal motility, with a low incidence of urinary retention. However, it is associated with increased opioid tolerance, severe withdrawal symptoms, and an extended duration of MV. Fentanyl (3 mcg/kg) reduces neonatal pain scores, effectively manages postoperative pain in neonates, and increases GH levels in preterm neonates on MV. Fentanyl is considered a rapidly acting type of opioid that produces adequate analgesia in neonates and could be used in postoperative pain in neonates as well as proper analgesia in ventilated neonates with effective response and wide safety profile. Remifentanil exhibits a greater analgesic potency compared to fentanyl while also possessing a shorter duration of action. Fentanyl may provide rapid analgesia and reduce pain during tracheal intubation in ventilated neonates, chest tube insertion in neonates with pneumothorax, surgical incisions, and invasive postoperative procedures. Fentanyl is regarded as the preferred analgesic for neonates due to its strong analgesic properties and reduced side effects, including lower incidences of hypotension, gastrointestinal motility issues, and urine retention compared to morphine. Inhaled fentanyl was found to be more effective than the intravenous form.

Naloxone functions as an opioid antagonist and is essential for counteracting potential neonatal respiratory depression induced by opioids. In order to minimize the side effects of fentanyl, it should be administered slowly, which can reduce chest wall or muscle rigidity and associated respiratory depression [72-79].

##### 1.5.1.2 Non-Opioid Drugs

**i-Acetaminophen (Paracetamol)**

Acetaminophen is an analgesic that has been utilized in newborns across various studies and neonatal centers. This is primarily utilized alongside other analgesics to minimize opioid consumption, particularly in postoperative pain scenarios. Parenteral acetaminophen decreases the quantity of opioids required postoperatively and is particularly beneficial in postoperative care to reduce opioid use or dosage. The primary side effect of acetaminophen is liver toxicity, which occurs only when administered in excessive doses. It is regarded as a safe and effective analgesic for neonates. Acetaminophen may be utilized for pain management following various procedures, including routine immunizations and circumcision.

In neonates, acetaminophen can be administered *via* various routes, including oral, rectal, and intravenous, with minimal side effects observed. Acetaminophen rarely causes hepatic or renal injury in newborns compared to its use in older children, and intravenous acetaminophen does not induce hypothermia in newborns.

In neonates, the clearance of acetaminophen is significantly slower than in older children, necessitating less frequent dosing *via* oral or rectal routes. The dosage may consist of oral administrations of 10 to 15 mg/kg every 6-8 hours or rectal doses of 20 to 25 mg/kg. The administration of IV acetaminophen in neonates may involve an initial loading dose of 20 mg/kg, followed by a maintenance dose of 7.5-10 mg/kg/6-8 hours. The total daily doses, depending on gestational age, are 20 - 30 mg/kg/day for those from 24-30 weeks, 35 to 50 mg/kg/day for those from 31 to 36 weeks, and 50 to 60 mg/kg/day for those from 37-42 weeks.

Acetaminophen is considered one of the most critical medications in neonates, serving both antipyretic and analgesic functions. The mechanisms of action of acetaminophen are characterized as a weak inhibitor of prostaglandin synthesis, along with the inhibition of chemoreceptors and spinal neurotransmitters related to nitrous oxide. Acetaminophen may cause liver injury; therefore, it is advisable to avoid its use in neonates with impaired liver function. Rectal administration of acetaminophen is optimal for postoperative or ventilated neonates unable to tolerate oral intake. The rectal route of administration is a significant method due to the high vascularization of the rectum, which enhances the absorption and bioavailability of acetaminophen when administered per rectum in neonates. Parenteral acetaminophen in neonates may be utilized in post-surgical pain protocols, often in conjunction with opioids for postoperative analgesia. This approach can mitigate the cumulative effects of opioid use, thereby reducing associated side effects. Preterm infants are more susceptible to opioid-related side effects. Therefore, when these neonates undergo painful procedures, the administration of parenteral acetaminophen is preferable. Acetaminophen exhibits a combination of safety and efficacy, rendering it a promising option for neonates and potentially decreasing the total morphine volume needed for the management of postoperative pain [80-91].

**ii-Non Steroidal Anti-Inflammatory Drugs (NSAIDs)**

The use of NSAIDs in neonates is restricted to medical closure due to their effects on renal function and their role in the development of neonatal pulmonary hypertension. Ketorolac is recognized as a strong inhibitor of prostaglandin synthesis. Ketorolac use may be associated with bleeding disorders in neonates, leading some neonatal centers to avoid its application for postoperative pain in newborns. However, other neonatal centers have administered ketorolac following cardiac surgery, 0.5 mg/kg/6 hours. Limiting the use of ketorolac in neonates is recommended, but further investigations and studies are pending to establish its safety and efficacy. Nonsteroidal anti-inflammatory drugs, such as indomethacin and ibuprofen, are primarily utilized for the management of PDA. Their mechanism of action involves the inhibition of cyclooxygenase enzymes (COX-1 and COX-2), which are responsible for the synthesis of prostaglandins. Therefore, limiting the use of NSAIDs in neonates is recommended; hence, further investigations and studies are required to establish their safety and efficacy, given the numerous side effects associated with these medications, such as renal impairment, platelet disorders, and neonatal pulmonary hypertension [92-96].

**iii-Dexmedetomidine**

Dexmedetomidine is a specific selective α2-agonist that induces analgesia while avoiding respiratory depression. The action mechanism involves the inhibition of substance P release. Dexmedetomidine may reduce opioid use in neonates. The route of administration of dexmedetomidine is IV or IM. Additionally, it can be administered orally, buccally, or intranasally. However, it is recommended to restrict the use of NSAIDs in neonates [97-100].

**iv-Benzodiazepines**

Benzodiazepines, such as midazolam, are primarily utilized in the NICU; however, their limited analgesic effect raises controversy regarding their application for analgesia in neonates. Numerous side effects associated with these drugs include respiratory depression, changes in blood pressure, and neurological impairment. Therefore, continuous monitoring of neonates receiving these medications is necessary. Midazolam is primarily utilized in ventilated neonates, particularly following surgery, demonstrating a positive impact on pain scores in this population. A loading dose of 100 mcg/kg, followed by a maintenance dose of 50 mcg/kg/hour, may be utilized during the neonatal period to achieve sedation. Lorazepam may also be utilized in the NICU, exhibiting a longer action duration than midazolam (6-12 hours) [101, 102].

**v-Chloral Hydrate**

Chloral hydrate reduces pain scores in neonates; however, it is indicated solely for sedation, not analgesia, and should be administered with extreme caution in neonates [103, 104].

**vi-Methadone**

Methadone can potentially be used for neonatal pain management; however, its use in neonates should be restricted, and further investigations and studies are required to establish safety and efficacy, given the potential for numerous side effects [105, 106].

**vii-Ketamine**

Ketamine is an anesthetic drug that may induce analgesia in neonates at low doses. However, its use in this population should be, and further research needs to be conducted to establish safety and efficacy, given the potential for adverse effects, such as neurotoxicity. Ketamine does not affect cerebral blood flow, making it a theoretically suitable drug for unstable neonates with altered blood pressure or hemodynamic instability during or after ventilation or surgical procedures, as it exerts minimal effects on cardiac function or blood pressure. The doses utilized in studies involving neonates are 1–2 mg/ kg/dose [107-109].

**viii-Propofol**

Propofol may be utilized for sedation; however, its application in neonates should be restricted, and further investigations and studies need to be conducted to establish safety and efficacy, given the numerous side effects and the absence of practical recommendations for its use in neonates [107-109].

**ix-Gabapentin**

Gabapentin inhibits the secretion of excitatory transmitter analgesics. Gabapentin may alleviate neonatal pain in the NICU and reduce opioid consumption due to its lower incidence of side effects compared to opioids. Nevertheless, further studies are required to validate its regular application for neonatal pain management [107-109].

**x-Local Anesthesia**

Neuraxial blockade can serve as an alternative to opioid analgesics for managing neonatal procedural and post-surgical pain, utilizing spinal or epidural blockade *via* the administration of local anesthetics. Local anesthetic agents, including lidocaine, are primarily utilized for regional anesthesia. Local anesthetic drugs reduce pain while minimizing the side effects associated with systemic medications, such as opioids. They facilitate earlier extubation and decrease respiratory complications. They may be utilized independently for minor procedures or surgeries, such as chest tube insertion and circumcision [107-109].

**xi-Topical Analgesics**

EMLA is a combination of two drugs, 2.5% prilocaine and 2.5% lidocaine, which, when applied to the skin, produces anesthesia at the application site. It results in a reduction of pain associated with lumbar puncture and venipuncture, particularly when combined with oral sucrose. Topical analgesics are effective in alleviating pain in neonates under various conditions, such as circumcision, particularly when used in conjunction with oral sucrose. Side effects include methemoglobinemia in addition to skin allergy, and preterm neonates are more susceptible to developing these side effects than full-term neonates [107-109].

#### 1.5.2 Non-Pharmacological Therapies or Environmental Measures

They include skin-to-skin care, oral sucrose, and facilitated tucking, as well as the therapy of non-nutritive sucking and breastfeeding. In the event of suspected neonatal meningitis, these therapies may be administered alone or in conjunction with other interventions to alleviate mild or moderate neonatal pain. These therapies include heel sticks, intravenous canulation or injections, neonatal circumcision, insertion of an orogastric tube, and neonatal lumbar puncture. Intelligent strategies are utilized to divert the attention of neonates and introduce alternative stimuli to reduce the pain transmitted to the brain by the desired intervention. For example, sensorial saturation, which involves the mother or the nurse acting the role of a conversational partner with the neonate prior to the desired procedure, may be implemented. The presence of the mother in any procedures performed on the neonates is advantageous, as is the use of skin-to-skin contact between the mother and her neonate or breastfeeding during procedures, such as sampling [110-115].

##### 1.5.2.1 Oral Sucrose/Glucose

The most significant and prevalent non-pharmacological approach to neonatal pain management is the administration of oral sucrose solution. Oral sucrose feeding is a straightforward bedside method for the treatment of neonatal pain. Oral feeding may alleviate neonatal pain by increasing the endogenous secretion of endorphins in the neonate. In addition to echocardiography and retinal examinations, oral sucrose or glucose has been demonstrated to be effective in simple procedures, such as venipuncture, insertion of an orogastric tube, heel stick, and IM injections. During mild or moderately painful interventions, oral sucrose provides neonatal analgesia. Sucrose is a safe and highly effective method for reducing neonatal pain resulting from a straightforward procedure. A significant reduction in pain scores was observed when sucrose was administered two minutes prior to a straightforward painful procedure, and the relief lasted for four minutes. To achieve complete analgesic effects, maneuvers that require a longer duration, such as circumcision, may necessitate multiple doses of sucrose. When sucrose is administered in conjunction with other techniques, such as non-nutritive sucking or swaddling, for painful procedures, such as routine immunizations and retinal examinations, a synergistic analgesic effect is observed. It was advised to administer an oral dose of 0.1 to 1 mL of 24% sucrose (or 0.2 to 0.5 mL/kg) two minutes prior to painful maneuvers. Glucose was demonstrated to be as effective as sucrose in reducing the response to mild to moderately painful procedures. In heel lancing and venipuncture, administering glucose solutions at a concentration of 20% to 30% reduces pain scores and a reduction in crying. Glucose may be administered as an alternative to sucrose solutions. However, prolonged procedures may not be advantageous for either glucose or sucrose. Gagging, coughing, vomiting, and hyperglycemia are all potential adverse effects of the consumption of either sucrose or glucose. Compared to a placebo, oral glucose administration in the form of 10% dextrose, breast milk, or both was effective in reducing neonatal pain during painful maneuvers, such as venipuncture or heel lancing. Moreover, 10% dextrose is a cost-effective and readily accessible solution for neonatal pain treatment in the NICU. It can be administered in a variety of painful maneuvers.

Administration of sucrose or glucose solutions to neonates two minutes prior to painful procedures stimulates the release of endogenous opioids, which exert intrinsic analgesic effects by inhibiting pain pathways. Sucrose or glucose solutions are associated with a reduction in crying time, tachycardia, and other pain scores. The administration of 0.5 ml of 24% sucrose, along with multiple doses, is more advantageous than a single dose for painful procedures. Sucrose at a concentration of 24% is beneficial for premature infants and is regarded as more effective than several glucose solutions. Sucrose is recognized as a safe and effective intervention for alleviating pain associated with procedures, such as venipuncture, intramuscular injection, or lumbar puncture. The administration of sucrose for neonatal pain management necessitates the use of the minimal effective dose for pain relief: 0.5 ml for neonates aged 27 to 31 weeks, 1 ml for those aged 32 to 36 weeks, and 2 ml for neonates 37 weeks or older. Administering small quantities of sucrose during painful procedures may elicit a sustained analgesic effect [116-125].

##### 1.5.2.2 Breastfeeding

Maternal breastfeeding has demonstrated efficacy in reducing pain responses in neonates during painful procedures. This effect is attributed to skin-to-skin contact, the act of sucking, and the sensory attributes of breast milk, including its odor and taste. Breastfeeding encompasses various components and is demonstrated to be a preferred method for pain management in neonates [126-131].

Breastfeeding is more effective in reducing pain in full-term neonates compared to preterm neonates. Breastfeeding may serve as an intervention for alleviating neonatal pain during procedures, such as heel sticks, intramuscular injections, and venipuncture. Breastmilk odor and taste have been shown to reduce pain in neonates and are beneficial during painful procedures, such as heel sticking. The combination of breastmilk odor and taste provides more significant analgesic effects and is more effective in alleviating neonatal pain during the heel stick procedure than alone. Thus, maternal breastfeeding is advantageous in reducing pain responses in neonates, as shown in Fig. (**2**) [126-131].

##### 1.5.2.3 Skin-to-Skin Care (SSC)

 It involves direct skin-to-skin contact between the newborn and the mother or caregiver. This contact was demonstrated to increase oxytocin secretion and decrease cortisol secretion following one hour of skin-to-skin contact. SSC alleviates the stress associated with neonatal pain during heel lancing, venipuncture, and, to a degree, minor surgical procedures. SSC during painful procedures reduces pain indicators and lowers pain scores in neonates. SCC effectively alleviates pain in neonates during acute procedures, such as capillary punctures. SSC should commence prior to the painful maneuver and persist throughout its duration to optimize its effectiveness in reducing the pain response of the neonate and enhancing the overall management of neonatal pain. Furthermore, SSC is highly effective in reducing pain responses in preterm neonates during painful procedures, such as heel puncture, compared to those who do not receive SSC during these interventions. It is recognized as an effective non-pharmacological intervention for managing and alleviating the pain response in both full-term and preterm neonates from thirty weeks of gestational age [132-139].

##### 1.5.2.4 Kangaroo Care (KC)

KC is classified as a type of SSC. KC diminishes crying duration, lowers pain scores, and alleviates stress in neonates during painful procedures. KC is demonstrated to be an effective form of SCC, and non-pharmacological interventions significantly reduce pain responses and enhance pain scores in neonates. The intervention may be safely administered to preterm neonates weighing over 1 kg, providing advantages for both mothers and their infants by reducing stress related to painful procedures in neonates [132-139].

##### 1.5.2.5 Swaddling

It is a common practice involving the gentle wrapping of a neonate in a lightweight, breathable blanket to promote a sense of calm and facilitate sleep. Recommendations suggest that individuals should wrap only their main body and avoid wrapping the neck or head to reduce the risk of suffocation. The swaddling maneuver is indicated for clinically stable neonates undergoing mild, simple, painful procedures. The consistent application of the swaddling maneuver to neonatal tactile receptors generates stimuli that may disrupt and compete with neonatal pain and mild to moderate stress, particularly when administered prior to and during painful procedures for neonates [132-139].

##### 1.5.2.6 Facilitated Tucking (FT)

FT is a technique that involves gently positioning a neonate’s upper and lower limbs in a flexed position close to the body, utilizing a blanket or nest. It is effective in painful procedures, resulting in preterm neonates crying for shorter durations with minimal alterations in neonatal heart rate. This maneuver’s mechanism of action may involve the neonatal posture providing persistent stimuli to the brain, potentially interfering with or competing against painful sensations by influencing pain perception. The application of FT in preterm neonates during painful procedures has been shown to reduce pain and enhance neonatal pain scores. It may be applied in minimal procedures, including heel sticking, venipuncture, and routine suctioning, primarily in preterm neonates. It may be utilized alongside other interventions, such as oral sucrose, to enhance the efficacy of FT. FT may be applicable in various minor painful procedures in neonates, including endotracheal or pharyngeal suction. FT is recognized for its safety and serves as a non-pharmacological intervention for pain management in neonates, potentially mitigating the side effects associated with pharmacological treatments for neonatal pain. FT involves limited mobility of the affected neonates, which can be preferred in ventilated neonates [132-139].

##### 1.5.2.7 Massage Therapy

It includes skin and soft tissue manipulation by the hands of the caregiver. The mechanism of action includes stimulation of vagal tone, modulation of insulin, and reduction in cortisol and adrenaline levels. Massage therapy showed a significant reduction of pain in neonates through vagal stimulation with subsequent enhanced neurological and developmental outcomes, especially in VLBW neonates [132-139].

##### 1.5.2.8 Non-Nutritive Sucking (NNS)

 It reduces neonatal response and reaction to pain during minor procedures in the NICU. In addition, it can be combined with breastfeeding and or to produce a synergistic analgesic effect, resulting in a significant and consistent reduction of pain in neonates, particularly preterm and LBW neonates, during minor painful maneuvers, such as heel sticking. The combination of oral sucrose and NNS may provide a more effective and sustained reduction in neonatal pain compared to the use of either method in isolation. The use of a pacifier or a sterilized gloved finger may alleviate neonatal pain and reduce discomfort in neonates. NNS reduces the intensity and duration of pain in neonates subjected to minor painful procedures. The mechanism of action involves enhanced oxygenation, improved respiratory and intestinal functions, and a decreased heart rate. NNS and FT result in reduced pain scores in neonates undergoing painful procedures, such as heel sticking, IM injections, and venipuncture [132-139].

##### 1.5.2.9 Ear Muffs and Eye Shield

 Ear muffs and eye shields effectively alleviate neonatal pain during painful procedures. The mechanism of action involves protection from environmental stimuli that may aggravate the sensation of pain, like light and sound. They are considered adequate, beneficial, easy, and relatively cheap methods for controlling and preventing neonatal pain, especially in minor painful maneuvers, such as venipuncture or routine blood sampling in any NICU [132-139].

##### 1.5.2.10 Endocrinal Changes in Response to Neonatal Pain

Neonatal pain results in decreased cortisol secretion and levels. Cortisol is a crucial hormone necessary for the functioning of all body organs and systems. Elevated oxytocin secretion is a critical hormone that reduces the activity of the hypothalamic-pituitary-adrenal (HPA) axis, regulates autonomic stress responses, attenuates inflammation, and decreases anxiety due to neonatal pain. The normalization of endocrinal hormones, such as cortisone and oxytocin, is facilitated by the management of neonatal pain, which affects their levels [140-142]. The common neonatal analgesic drugs and their dosing are illustrated in Table **1** [142-148].

## CONCLUSION

Neonates respond to pain through a variety of physiological, emotional, and endocrine responses, such as changes in cortisone and oxytocin serum levels, as well as physiological and emotional reactions. The management of neonatal pain results in the normalization of endocrine hormones, such as cortisone and oxytocin, which are previously affected by neonatal pain. Neonates experience pain and exhibit reactions similar to those of other age groups. Newborns experience pain; however, their lack of expression does not indicate an absence of pain perception. Unmanaged pain in neonates can lead to various sequelae, including short-term effects, such as increased heart rate and crying, which occur immediately following pain exposure. Furthermore, long-term sequelae may influence neurodevelopmental and physical outcomes. Numerous scores exist for the assessment of neonatal pain; however, consensus on a single validated score is lacking. Many neonatal units have not prioritized neonatal pain management and lack established protocols for its assessment and treatment. Various treatment modalities for neonatal pain exist, including opioid medications, such as morphine, non-opioid agents like acetaminophen, and non-pharmacological interventions, such as the administration of sucrose solution. We recommend further studies on the efficient and safe treatment measures of neonatal pain and worldwide acknowledgment for all medical staff working regarding the significance of assessing and managing neonatal pain.

## Figures and Tables

**Fig. (1):**
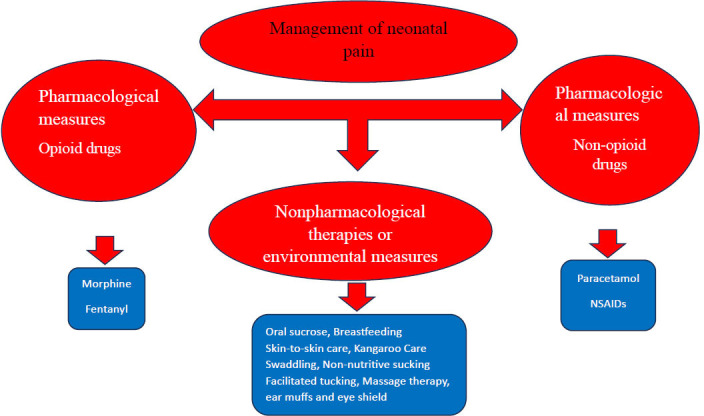
The management of neonatal pain.

**Fig. (2):**
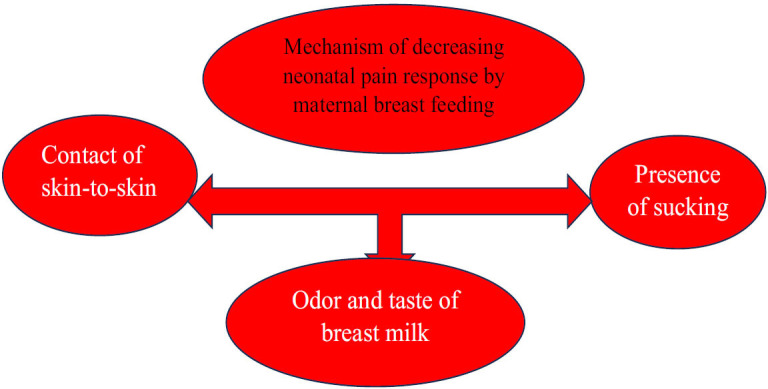
Mechanism of decreasing neonatal pain response by maternal breastfeeding.

**Table 1:** Common neonatal analgesic drugs and their dosing.

**Drug**	**Dose and Administration**
**Opiates** **Morphine** **Fentanyl**	IV: 0.05 mg per kg per dose IM or IV: 0.5 to 1 mcg per kg per dose Intranasal: 1.5 to 2 mcg per kg per dose
**Acetaminophen**	Oral route: 10 mg per kg and given every 6 h Rectal route: 20 mg per kg IV: 10 mg per kg at first, and then 50 mg per kg per day
**Sucrose/Glucose**	Oral: 20–30% solution,
**Local anesthetic** **EMLA (2.5% lidocaine in addition to 2.5% prilocaine)** **Lidocaine injection**	Topical, controversial Subcutaneous or IM, controversial

**Note: ** [142-148].

## LIST OF ABBREVIATIONS

AD: Anesthetic Drugs
EH: Endocrine Hormones
HPA: Hypothalamic Pituitary Adrenal
HR: Heart Rate
PSH: Primary Stress Hormone
